# Fetal Growth Restriction and Long-Term Cardiovascular Morbidity of Offspring in Dichorionic–Diamniotic Twin Pregnancies

**DOI:** 10.3390/jcm12041628

**Published:** 2023-02-17

**Authors:** Tuval Tzafrir, Tamar Wainstock, Eyal Sheiner, Shayna Miodownik, Gali Pariente

**Affiliations:** 1 Joyce and Irving Goldman Medical School, Faculty of Health Sciences, Ben-Gurion University of the Negev, Beer-Sheva 8410101, Israel; 2 Department of Public Health, Faculty of Health Sciences, Ben-Gurion University of the Negev, Beer-Sheva 8410501, Israel; 3 Department of Obstetrics and Gynecology, Soroka University Medical Center, Ben-Gurion University of the Negev, Beer-Sheva 8410101, Israel

**Keywords:** long-term cardiovascular morbidity, diamniotic–dichorionic twin pregnancies, fetal growth restriction (FGR), offspring

## Abstract

**Objective:** We opted to investigate whether fetal growth restriction (FGR) in dichorionic–diamniotic twins is a risk factor for long-term cardiovascular morbidity in offspring. **Study design:** A population-based retrospective cohort study, comparing long-term cardiovascular morbidity among FGR and non-FGR twins, born between the years 1991 and 2021 in a tertiary medical center. Study groups were followed until 18 years of age (6570 days) for cardiovascular-related morbidity. A Kaplan–Meier survival curve compared the cumulative cardiovascular morbidity. A Cox proportional hazard model assisted with adjusting for confounders. **Results:** In this study, 4222 dichorionic–diamniotic twins were included; 116 were complicated with FGR and demonstrated a significantly higher rate of long-term cardiovascular morbidity (4.4% vs. 1.3%, OR = 3.4, 95% CI 1.35–8.78, *p* = 0.006). The cumulative incidence of long-term cardiovascular morbidity was significantly higher among FGR twins (Kaplan–Meier Log rank test *p* = 0.007). A Cox proportional-hazard model found an independent association between FGR and long-term cardiovascular morbidity, when adjusted for both birth order and gender (adjusted HR 3.3, 95% CI 1.31–8.19, *p* = 0.011). **Conclusions:** FGR in dichorionic–diamniotic twins is independently associated with an increased risk for long-term cardiovascular morbidity in offspring. Therefore, increased surveillance may be beneficial.

## 1. Introduction

The prevalence of twin pregnancies has increased significantly over the past few decades, reaching approximately 3 percent of live births in the United States [1]. Approximately one-third of the increase in multiple births in recent decades can be attributed to an increasing age at childbirth [2]. Older individuals are more likely to utilize fertility treatments, which substantially increase the prevalence of twin pregnancy when compared to that with natural conception [3]. Over one-third of all twin infants born in the United States is attributed to the use of assisted reproductive technologies (ARTs) [4].

Twin pregnancies have been associated with an increased risk for adverse pregnancy and neonatal outcomes. Gestational hypertension, preeclampsia [5], gestational diabetes mellitus [6], selective fetal growth restriction (FGR), preterm delivery, cesarean delivery, and post-partum hemorrhage (PPH) are all associated with twin pregnancies [7,8]. Additional maternal disorders observed more often in women pregnant with multiple gestations include acute fatty liver of pregnancy, intrahepatic cholestasis of pregnancy, hyperemesis gravidarum, iron deficiency anemia, and thromboembolism [9].

FGR is defined as less than normal fetal growth in which the estimated weight of the fetus is below the 10th percentile [8]. FGR infants have been found to have a higher risk of both immediate adverse perinatal outcomes, such as prematurity, impaired thermoregulation, hypoglycemia, and impaired immune function [10], as well as long-term morbidities, such as neurodevelopment abnormalities [11], respiratory [12], and cardiovascular morbidity [13].

Studies have associated placental-related complications, such as preeclampsia and placental abruption, with long-term maternal and child cardiovascular morbidity [14,15]. As FGR is considered to be a part of the placental-associated syndrome [16] and due to its high prevalence among twin pregnancies, we opted to compare the association of long-term cardiovascular morbidity between dichorionic–diamniotic twins presenting with and without FGR. Understanding the relationship between FGR in twins and cardiovascular morbidity will contribute to early diagnosis of those offspring. 

## 2. Materials and Methods

### 2.1. Study Design

A population-based retrospective cohort study was conducted. The main exposure was defined as FGR in dichorionic–diamniotic twins, present in either one or both fetuses in each dichorionic–diamniotic twin pregnancy. The main outcome was defined as long-term cardiovascular morbidity up to 18 years (6570 days). Cardiovascular morbidity was assessed using a predefined set of ICD-9 codes associated with hospitalization of the child. Cardiovascular morbidity included the following: structural valvular diseases, such as tricuspid, mitral, pulmonary, and aortic valve disease, hypertension, arrhythmia, rheumatic fever, pulmonary heart disease, peri-myo-endocarditis, ischemic heart diseases, cardiomyopathy, congenital heart defect, diastolic heart failure, and total cardiovascular hospitalizations. Only the first hospitalization per diagnosis for each child was included in the analysis. 

### 2.2. Setting

The study was conducted at the Soroka University Medical Center (SUMC), the sole tertiary hospital in the southern region of Israel, serving over 1 million people, with 15,000 to 17,000 deliveries annually. This study was approved by the institutional review board in accordance with the Helsinki declaration (IRB number 0357-19-SOR). 

### 2.3. Study Population

All diamniotic–dichorionic twins with different genders born between the years 1991 and 2021 at SUMC were included. Twins with congenital malformations or chromosomal abnormalities were excluded from the study. Same-sex twins were also excluded from the study in order to verify that the study population included only dichorionic–diamniotic twin pregnancies. Cases of perinatal mortality or those with missing data were excluded from the long-term analysis.

### 2.4. Data Collection Method

Data were collected from two databases that were cross-linked and merged: the computerized perinatal database of the Obstetrics and Gynecology department and the computerized hospitalization database of Soroka University Medical Center (“Demgo-ICD9”). The obstetrical database includes demographic information, perinatal assessments, maternal morbidities, and maternal and fetal outcomes and complications and is recorded immediately following delivery by the attending physician and examined by skilled medical secretaries prior to being entered into the computerized database. Records were anonymized prior to analysis. The pediatric hospitalization database includes demographic and medical information, which is sorted by the international classification of diseases, ninth revision codes (ICD-9). Since follow-up was assessed through hospitalization records and no information was collected regarding immigration or community treatment, there was no way to distinguish between loss to follow-up or missing data and offspring without cardiovascular morbidity. Therefore, no information about missing data can be provided. The certainty of the relationship between mother and offspring is assured by the national security number (ID), which is attached to the offspring and registered in the mother’s formal identification card.

### 2.5. Study Size

Due to the nature of the retrospective-based study, the sample size was given. After excluding the offspring that did not meet the inclusion criteria, there were 116 children born from dichorionic–diamniotic pregnancies with suspected FGR, as well as 2106 children who were born from dichorionic–diamniotic pregnancies without a suspicion of FGR. Furthermore, the first analysis of the data revealed the assessed rate of pediatric cardiovascular hospitalizations in dichorionic–diamniotic twins to be 1/3%. 

Therefore, as the sample size was given, using the Winpepi software, the size effect was calculated, so that there was a power of 80%. This sample size has a power of 80% to detect an odds ratio of 4.7 between the study groups.

### 2.6. Statistical Analysis

Univariable analysis was performed to compare dependent characteristics between the two study groups: the exposed group of growth-restricted twins and the unexposed group of non-growth-restricted twins. The univariable analysis included the chi-squared test for categorical variables and *t*-test for continuous variables, according to their distribution. The categorical variables in this study included ethnicity, gravidity, parity, pregnancy following fertility treatment (ovulation induction or in vitro fertilization), maternal hypertensive disorders, maternal diabetes mellitus, preterm delivery, cesarean delivery, gender, low Apgar scores, perinatal mortality, and cardiovascular diagnoses. Continuous variables in this study included maternal age and birthweight. A Kaplan–Meier survival curve was used to compare the cumulative morbidity incidence between the groups, using the log-rank test to determine significant differences. A Cox proportional hazard model was used to control for potential confounders and for siblings. All analyses were performed using SPSS (version 26 or higher) or STATA software (version 16.0).

## 3. Results

**Figure 1:**
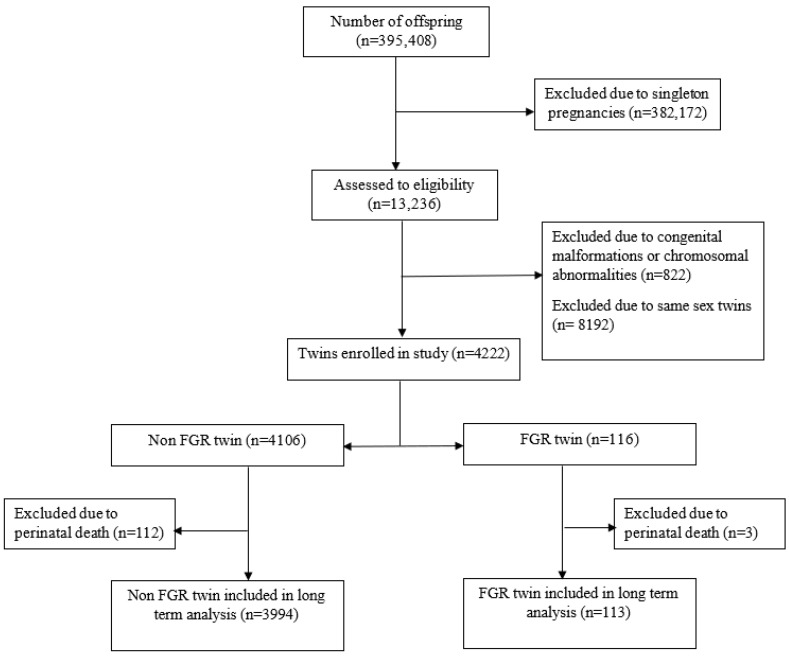
Number of individuals at each stage of the study.

Figure 1 demonstrates the number of individuals at each stage of the study. The study included 13,236 pairs of twins assessed for eligibility, of which, 822 were excluded due to congenital malformations or chromosomal abnormalities and 8192 were excluded due to same-sex twins, in order to verify that the study group included only dichorionic–diamniotic twin pregnancies. Therefore, during the study period, 4222 dichorionic–diamniotic twin newborns were included, of which, 116 were complicated with FGR in one or both twins. The mean follow-up time was 3976 ± 2309 days. Table 1 demonstrates the demographic characteristics of the study population, together with its perinatal outcomes. Mothers of twins presenting with FGR had significantly lower parity and were more likely to conceive following infertility treatments. In addition, they demonstrated higher rates of hypertensive disorders (25.9% vs. 10.0%, *p* < 0.001), preterm delivery (78.9% vs. 53.8%, *p* < 0.001), and cesarean delivery (72.4% vs. 55.1%, *p* < 0.001). For each variable, there was fewer than 5% missing values. Replacing the missing values was not conducted as there were only a few variables as such.

**Figure 2:**
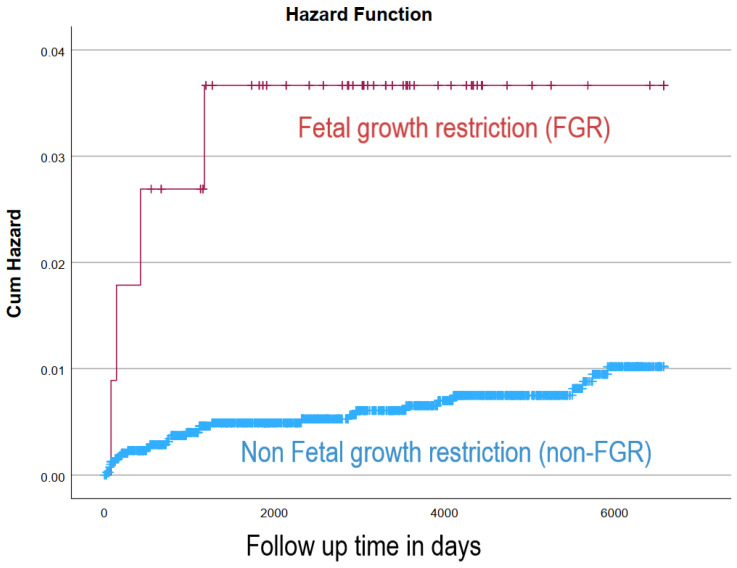
Cumulative incidence of long-term cardiovascular morbidity of the offspring according to fetal growth restriction in dichorionic–diamniotic twins.

After excluding all cases of perinatal mortality, the study included 4107 dichorionic–diamniotic twins, of which, 113 were complicated with FGR. Table 2 demonstrates the association between FGR and long-term cardiovascular morbidity of the offspring. Total cardiovascular hospitalizations were significantly higher among FGR twins (4.4% vs. 1.3%, *p* = 0.006) and specifically included structural valvular disease (0.9% vs. <0.1%, *p* = 0.001), childhood hypertension (1.8% vs. <0.1%, *p* < 0.001), pulmonary heart disease (0.9% vs. 0.1%, *p* = 0.006), and ischemic heart disease (0.9% vs. <0.1%, *p* = 0.001). The cumulative incidence of long-term cardiovascular morbidity was significantly higher among FGR twins (Kaplan–Meier survival curve Log rank *p* = 0.007, Figure 2). In the Cox proportional hazard regression model, an independent association was found between FGR and long-term cardiovascular morbidity, after adjusting for birth order and gender (adjusted HR 3.3, 95% CI 1.31–8.19, *p* = 0.011, Table 3). After excluding all cases of possible congenital valve anomalies, the independent association between growth restriction in dichorionic–diamniotic twins and long-term cardiovascular morbidity remained (adjusted HR 5.096, 95% CI 1.79–14.5, *p* = 0.002).

## 4. Discussion

The main finding of our study was the independent association between FGR in dichorionic–diamniotic twin pregnancies and long-term cardiovascular morbidity. Specifically, FGR among twins was associated with diagnoses, such as structural valvular disease, childhood hypertension, pulmonary heart disease, and ischemic heart disease. 

Previous studies have shown an association between FGR or small for gestational age (SGA) offspring and cardiovascular morbidity. However, to the best of our knowledge, no study explored this association in twin pregnancies. Barker et al. [17] have shown that reduced fetal growth is followed by increased mortality from cardiovascular disease in later years. It is important to distinguish that their study followed deaths from cardiovascular reasons in adulthood while we assessed cardiovascular morbidity by cumulative hospitalizations due to cardiovascular diagnoses during childhood. Furthermore, their group did not evaluate the effect of multifetal gestations. Agata et al. [18] discussed the association between SGA and hypertension in children six-to-ten-years-old and found that prehypertension or hypertension were more frequent in children who presented with SGA [18]. Our study analyzed a broader range of ages, as we followed the patients until the age of 18 years (6570 days) with a wider set of cardiovascular-related diagnoses. Further studies on singleton pregnancies with FGR have shown a correlation with long-term cardiovascular [13], neurologic [19], and endocrine [20] morbidities.

The underlying mechanism through which growth restriction increases the risk of cardiovascular morbidity may be related to the fetal programing theory. This theory states that the combination between the genotype and intrauterine environment plays a significant role in the development of disease later in life [14]. Growth restriction can occur due to a lack of nutrients or oxygen in fetal life. Placental-associated diseases, such as preeclampsia, growth restriction, or placenta abruption, may cause placental malperfusion and through that enhance the downregulation of protein kinase B or AKT/mTOR (rapamycin), a nutrient-sensing pathway that affects the nutrient supply to and through the placenta [18]. AKT/mTOR acts as a translation regulator and oversees mRNA translation into protein. AKT/mTOR also regulates the expression of placental transporters, which in turn are responsible for transporting nutrients, such as fatty acids, amino acids, and glucose, into placental cells. By doing so, AKT/mTOR affects the placental surface by reducing the exchange surface area and in turn the number of transporters [18]. In the case of undernutrition, the fetus may adapt by slowing the cell replication rate, which transcribes into growth restriction, as this is the main method by which the embryo increases in size. This adaptation occurs directly by the cells reacting to the lack of nutrients and indirectly by changing the concentration of different growth hormones. This process may affect the sum number of functioning cells in an organ and the distribution of cell types. Therefore, undernutrition may cause irreversible changes in the embryonic cardiovascular system, which may predispose these individuals to cardiovascular morbidity later in life [21]. Additionally, there are higher rates of growth restriction seen in twin over singleton pregnancies [22]. This may be a result of failure of the utero-placental units to supply the nutritional needs of both embryos, physical restraint imposed by the uterine size and wall, or a primary placental growth restriction due to the presence of another placenta in dichorionic twin pregnancies [22]. These mechanisms may increase the severity of undernutrition in twin pregnancies and lead to the lack of proper organ development. Nevertheless, the specific mechanisms that take place and result in undernutrition and subsequently cardiovascular susceptibility later in life have yet to be defined. This could be a result of programing of the immune system, antioxidant defenses, inflammatory responses, stem cell quality, the neuroendocrine system, and the autonomic nervous system, which have all been demonstrated to be associated with cardiovascular morbidity [23]. 

Our study’s major strength stems from the fact that SUMC is the sole tertiary hospital in the southern region of Israel. That, combined with free essential health insurance provided to each citizen of Israel, makes it safe to assume that if a woman gave birth to a child in SUMC, the child would reach SUMC when in need of major medical assistance. Our study does have several limitations. First, the database used for this study includes only children who were admitted to the hospital and not ambulatory cases. Hence, children with mild-to-moderate cardiovascular morbidity that did not require hospitalization would not be included as events. A possible solution may be to conduct a sequel study, which will align the community-acquired medical records with the hospitalization database. Second, the study used intra-uterine growth estimation, which is an estimation of the fetal weight, rather than the real weight of the newborn. Following standard protocols of estimated weight using local growth curves, together with skilled ultrasound end-users, may minimize the difference between the estimated growth restriction and the actual small weight after birth. Third, coding mistakes are always a possibility in a retrospective study design. Nevertheless, all the data were examined by skilled medical secretaries before being entered into the computerized database. Fourth, immigration and thus loss to follow-up may also be a possible weakness of this study. However, negative or positive immigration may affect both groups in a similar manner. Fifth, there may be a higher incidence of genetic abnormalities in the FGR twins that puts them at a higher risk for long-term cardiovascular morbidities. While it still would support the association between FGR twins and long-term cardiovascular morbidity, the pathophysiology may be different. Nevertheless, twins with diagnosed congenital malformations or chromosomal abnormalities were excluded from this study. Sixth, the higher risk for long term cardiovascular morbidity may not be strictly related to growth restriction. For example, childhood ischemic heart disease may suggest an acquired post-infectious coronary artery abnormality rather than cardiovascular morbidity related to FGR. Seventh, prematurity, a possible independent risk factor for cardiovascular morbidity [24], was not evaluated in this study due to the small number of prematurity cases. Lastly, since the prevalence of childhood cardiovascular morbidity is low, the study groups did not include many events. Therefore, the number of occurrences of long-term cardiovascular morbidity is quite small, with one or at most two events of a particular cardiovascular morbidity in the FGR group. More studies with larger study groups may be needed. 

In conclusion, our research has shown that FGR in dichorionic–diamniotic twins is independently associated with an increased risk of long-term cardiovascular morbidity of the offspring. FGR offspring born from diamniotic–dichorionic twin pregnancies may benefit from increased surveillance, although further research is needed before certain recommendations can be provided. Understanding the relationship between FGR in twins and cardiovascular morbidity may contribute to early diagnosis and treatment. Additional surveillance may possibly lead to early diagnosis and improve the future health of FGR twins. Additional studies are required to understand the specific mechanism that causes FGR twin offspring to be more susceptible to long-term cardiovascular morbidity. 

## Figures and Tables

**Table 1:** Demographic characteristics of study population and perinatal outcomes.

Characteristics		Mother to FGR Twin (n = 116) N (%)	Mother to Non-FGR Twin (n = 4106) N (%)	*p* Value
Maternal age, years (mean + SD),		30.74 + 6.4	29.95 + 5.5	0.125
gravidity	1	38 (32.8%)	1034 (25.2%)	0.179
	2–4	48 (41.4%)	1866 (45.4%)	
	5+	30 (25.9%)	1206 (29.4%)	
Parity	1	52 (44.8%)	1312 (32.0%)	0.011
	2–4	46 (39.7%)	1888 (46.0%)	
	5+	18 (15.5%)	906 (22.1%)	
Pregnancy following fertility treatment		48 (41.4%)	1226 (29.9%)	0.008
Hypertensive disorders *		30 (25.9%)	412 (10.0%)	<0.001
Diabetes mellitus **		8 (6.9%)	394 (9.6%)	0.329
Preterm delivery		90 (78.9%)	2208 (53.8%)	<0.001
Cesarean delivery		84 (72.4%)	2261 (55.1%)	<0.001
Gender	Male	58(50.0%)	2053 (50.0%)	1.00
	Female	58 (50.0%)	2053 (50.0%)	
Mean birthweight, gr (mean + SD)		1904.69 + 525.0	2339.79 + 555.9	<0.001
5th minute Apgar score <7		1 (0.9%)	88 (2.2%)	0.519
Perinatal mortality		3 (2.6%)	112 (2.7%)	0.926

* Hypertensive disorders: chronic hypertension, gestational hypertension, and preeclampsia; ** Diabetes mellitus: pre-gestational and gestational diabetes mellitus.

**Table 2:** Comparison of select long-term cardiovascular morbidity of dichorionic–diamniotic twins stratified by FGR.

	Dichorionic–Diamniotic Twin with FGR (n = 113) N (%)	Dichorionic–Diamniotic Twin without FGR (n = 3994) N (%)	OR	95% CI	*p* Value
Structural valvular disease	1 (0.9%)	2 (0.1%)	17.8	1.60–197.98	0.001
Childhood hypertension	2 (1.8%)	2 (0.1%)	36	5.02–257.63	<0.001
Arrhythmia	0 (<0.1%)	13 (0.3%)	1	0.99–1.00	0.544
Rheumatic fever	0 (<0.1%)	1 (<0.1%)	1	0.99–1.00	0.866
Pulmonary heart disease	1 (0.9%)	3 (0.1%)	11.9	1.23–115.08	0.006
Peri-, myo-, endocarditis	0 (<0.1%)	4 (0.1%)	1	0.99–1.00	0.736
Ischemic heart diseases	1 (0.9%)	2 (<0.1%)	17.8	1.60–197.98	0.001
Diastolic heart failure	0 (<0.1%)	1 (<0.1%)	1	0.99–1.00	0.866
Cardiac heart disease, not-otherwise specified	0 (<0.1%)	3 (0.1%)	1	0.99–1.00	0.771
Total cardiovascular hospitalizations	5 (4.4%)	53 (1.3%)	3.4	1.35–8.78	0.006

**Table 3:** Cox proportional hazards model to predict offspring long-term cardiovascular morbidity.

Variables	Adjusted HR	95% CI	*p* Value
FGR dichorionic–diamniotic twin (vs. non-FGR dichorionic–diamniotic twin)	3.3	1.31–8.19	0.011
Order of birth	1.3	0.79–2.23	0.289
Gender	1.1	0.64–1.80	0.784

## Data Availability

Data is unavailable due to Institutional Review Board (IRB) consideration.

## Abbreviations

Fetal growth restriction (FGR), assisted reproductive technologies (ART), Soroka University Medical Center (SUMC), small for gestational age (SGA).
